# Rapid preliminary screening of Tomato brown rugose fruit virus based on surface-enhanced Raman spectroscopy and machine learning

**DOI:** 10.3389/fpls.2026.1866144

**Published:** 2026-06-10

**Authors:** YongDe Xu, RongXia Huang, FuJian Ren, JinFei Lin, Jing Zhang, YuShi Jiang

**Affiliations:** 1 School of Electromechanical Engineering, Guangdong University of Technology, Guangzhou, China; 2 State Key Laboratory for High Performance Tools, Guangdong University of Technology, Guangzhou, China; 3 Key Laboratory of Advanced Materials of Ministry of Education, School of Materials Science and Engineering, Tsinghua University, Beijing, China; 4 Qingxin Zhipu (Foshan) Technology Co., Ltd., Foshan, Guangdong, China

**Keywords:** label-free biosensing, machine learning, rapid screening, SERS, Tomato brown rugose fruit virus

## Abstract

**Introduction:**

Tomato brown rugose fruit virus (ToBRFV) represents a growing threat to global tomato production, causing severe losses in crop yield and fruit quality. Although the standard RT-qPCR assay is highly accurate, its reliance on laboratory processing, specialized equipment, and trained personnel limits its applicability for rapid on-site diagnostics. To address this limitation, this study evaluated a biosensing method that does not require labels and combines surface-enhanced Raman scattering (SERS) with machine learning to distinguish tomato leaves infected with ToBRFV from healthy leaves.

**Methods:**

Following health status confirmation via RT-qPCR, leaf extracts were directly deposited onto silver nanorod arrays for SERS spectral acquisition. Three classification models were evaluated: PCA-LDA, PLS-DA, and SVM.

**Results:**

The results showed that all models were able to discriminate infected samples from healthy samples in the present dataset. Notably, the SVM model exhibited the best performance, achieving an accuracy of 91.67%, a sensitivity of 100.00%, a specificity of 81.48%, and an area under the ROC curve (AUC) of 0.993.

**Discussion:**

This result suggests that SERS spectra may contain biochemical information associated with ToBRFV infection and that such information can be used for sample classification using machine learning models. In its present form, this approach is intended as a rapid, low-cost, field-deployable preliminary screening tool — not a replacement for RT-qPCR or other confirmatory molecular assays. The reported accuracy was obtained on mechanically inoculated plants of a single cultivar under controlled greenhouse conditions and should therefore be interpreted as a proof-of-concept upper bound; field-scale validation is the focus of ongoing work.

## 1 Introduction

Tomatoes are one of the most valuable vegetable crops in the world. In recent years, Tomato brown rugose fruit virus (ToBRFV) has posed a serious threat to the tomato industry. This virus was first reported in greenhouse tomatoes in Jordan and was later identified in southern Israel (Salem et al., 2016). It is now regarded as one of the most serious threats to global tomato production. Based on estimates derived from data from Florida, USA, a ToBRFV outbreak in the state tomato crop could result in a potential yield loss of 30% to 70%, corresponding to an annual economic impact of approximately USD 262 million (Oladokun et al., 2019; Klap et al., 2020).

ToBRFV is an RNA virus belonging to the genus *Tobamovirus* and causes severe damage to crops such as tomato and pepper. Compared with related viruses, ToBRFV requires particular attention because it can overcome the resistance conferred by the Tm-2² gene. For decades, Tm-2² conferred robust protection against Tobacco mosaic virus and Tomato mosaic virus, safeguarding global tomato production. However, ToBRFV has overcome the resistance mediated by this gene. Furthermore, the rapid spread of the virus has resulted in substantial agricultural losses within a short period. To date, the virus has been detected across all major continents where tomatoes are cultivated. Even plants carrying the Tm-2² gene can develop severe disease symptoms, including mosaic leaf patterns, dead fruit spots, and rough wrinkles (Luria et al., 2017).

ToBRFV spreads primarily through mechanical contact and infected seeds and exhibits high environmental stability and infectivity. The virus can stick to the seed coat or endosperm, and this helps it travel long distances. Contaminated farm tools also spread the disease (Fidan et al., 2024). These features make prevention and control very difficult. Current detection methods mainly rely on molecular tests like RT-PCR and RT-qPCR. These tests are sensitive and reliable, but they require lab equipment, trained staff, and long processing times (Panno et al., 2019; Devi et al., 2024). Therefore, they are difficult to implement for rapid on-site screening in agricultural settings. Previous studies by Mandrile et al. demonstrated that Raman spectroscopy can be used for the early identification of *Tomato yellow leaf curl Sardinia virus* (TYLCSV) and *Tomato spotted wilt virus* (TSWV) in tomato plants (Mandrile et al., 2019). By contrast, few studies have applied Raman spectroscopy or SERS to the detection of ToBRFV in plant samples. Sacco et al. combined dielectrophoresis with Raman spectroscopy, and they found specific spectral patterns of ToBRFV in liquid suspensions (Sacco et al., 2023). Recent work indicates that Raman spectroscopy can provide chemically selective information for detecting plant diseases and physiological stress under greenhouse and field-related conditions. Sanchez et al., for instance, showed that Raman spectroscopy coupled with a PLS-DA model allowed early, non-destructive diagnosis of nutrient deficiency and salinity stress in rice before visible symptoms developed (Sanchez et al., 2020a). Furthermore, this technique induces minimal sample damage and does not require chemical labels (Farber and Kurouski, 2022).

**Figure 1:**
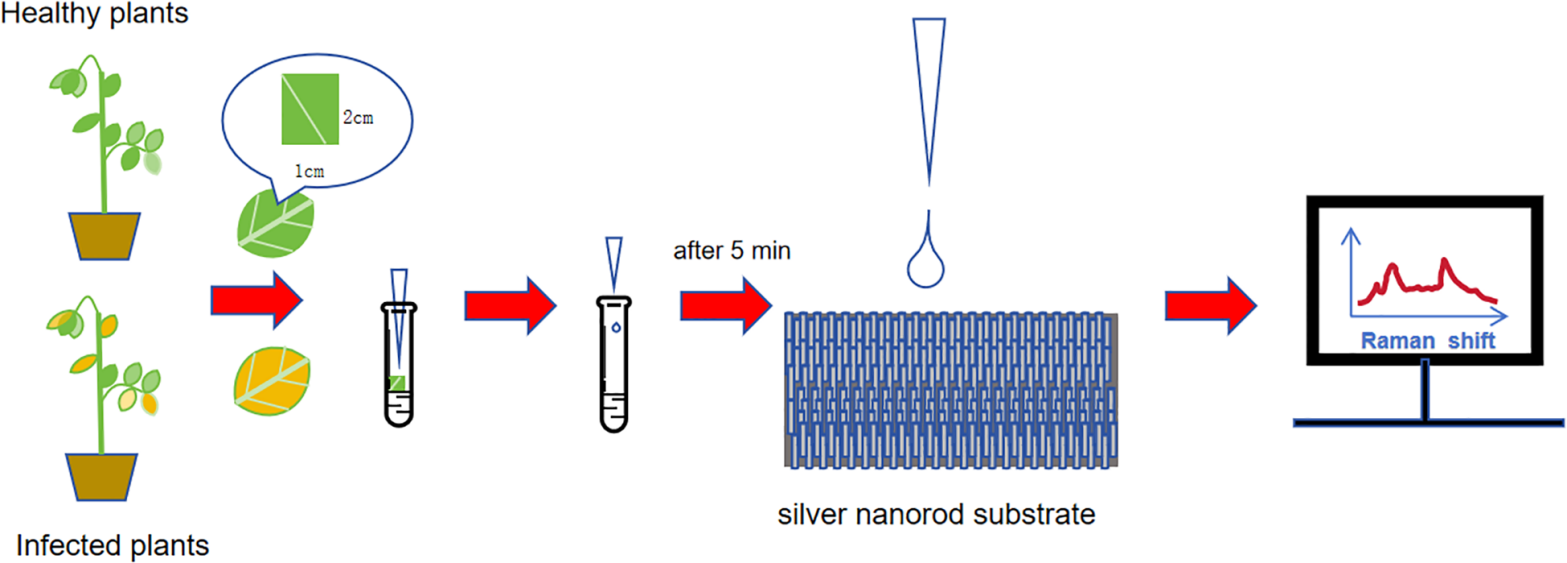
Experimental workflow for rapid preliminary ToBRFV screening. Tomato leaves were first extracted with ethanol and then diluted. The resulting samples were analyzed by SERS on silver nanorod arrays, followed by spectral preprocessing and the development of classification models based on PCA-LDA, PLS-DA, and SVM.

Internal biochemical changes often precede the appearance of visible symptoms in leaves and fruits. Because Raman spectra have the potential to capture biochemical changes that may precede or accompany visible symptoms, this approach may provide a basis for future early-stage screening studies, provided that further validation is performed using asymptomatic, early-infection, and field-collected samples. However, conventional Raman signals are inherently weak. Therefore, surface-enhanced Raman scattering (SERS) has been utilized as a more sensitive alternative. When combined with substrates such as silver nanorods, SERS has enabled rapid, label-free detection of biological targets, including pathogens such as rotavirus (Driskell et al., 2010; Wang et al., 2025b; Zhang et al., 2025). On this basis, the present study examined the use of SERS for preliminary screening of ToBRFV infection. By comparing SERS spectra from healthy and infected tomato plants under controlled experimental conditions, we assessed whether SERS combined with machine learning could support rapid preliminary screening for ToBRFV infection. The overall experimental workflow is summarized in **Figure 1**.

## 2 Materials and methods

### 2.1 Plant samples

Tomato plants were grown at 25 ± 2 °C until they reached the stage with four true leaves. At this stage, a subset of plants was mechanically inoculated with ToBRFV, while the remaining plants were maintained as non-inoculated healthy controls. Following inoculation, the control plants continued to grow normally and showed flat leaves with uniform coloration, whereas the inoculated plants gradually developed typical virus-associated symptoms, including leaf yellowing, mosaic patterns, and blistering. At 14 days post-inoculation, all plants were tested by RT-qPCR to confirm their infection status. After RT-qPCR verification, fresh leaf samples were collected from both infected and healthy plants for subsequent SERS analysis.

### 2.2 Preparation of SERS substrates

Silver nanorod array substrates were used as the SERS-active platform in this study. They were prepared by the glancing angle deposition (GLAD) method reported by Ma et al. GLAD, a variant of oblique angle deposition (OAD), is a well-established physical vapor deposition technique for producing large-area Ag nanorod array substrates with high uniformity. By adjusting parameters such as the incident angle and deposition rate, this method allows precise control of nanorod morphology and SERS performance.

The substrates were fabricated with an electron-beam evaporation system (Thermionics Laboratory Inc., USA). Silicon wafers with a <100> crystal orientation served as the supporting substrates and were rinsed with deionized water before deposition. Silver was used as the source material, and the chamber was evacuated to a base pressure of 5 × 10⁻^5^ Pa. The sample stage was then tilted to 86°, and the deposition thickness was set at 1000 nm. Detailed data on the morphology and SERS enhancement properties of this substrate have been reported elsewhere and are not repeated here. After deposition, the silver nanorod array-coated silicon wafers were cut into 1 cm × 1 cm pieces and used in the subsequent experiments (Chaney et al., 2005; Ma et al., 2015, 2017).

### 2.3 Pretreatment of leaf samples and Raman spectra acquisition

Healthy and infected leaves were cut into small pieces measuring 1 cm × 2 cm. These leaf fragments were placed in 1.5 mL centrifuge tubes containing 1 mL of ethanol. The mixtures were crushed using a pipette tip, followed by a 5-minute incubation at room temperature. The supernatant was collected, diluted 100-fold with ethanol, and thoroughly mixed. This dilution step minimized matrix effects from the plant extracts, improved the signal-to-noise ratio, and reduced molecular interference (Luo et al., 2018).

Subsequently, a 3 μL aliquot of the diluted extract was deposited onto the silver nanorod SERS substrate. The droplet was allowed to dry naturally at room temperature, during which target molecules adsorbed onto the substrate surface to generate stable SERS signals.

SERS spectra were acquired with a portable Raman spectrometer (BW TEK, BWS465-785S) controlled by BWSpec software and equipped with a 785 nm laser. Measurements were performed with a laser power of 50 mW, an integration time of 10 s, and a 20× objective over a spectral range of 100–2000 cm⁻¹. Spectra were collected from 48 healthy tomato plants and 49 ToBRFV-infected plants. For each sample, three spectra were acquired from distinct positions on the SERS substrate to reduce variability caused by local differences in substrate enhancement and measurement position, thereby improving the stability and reliability of the spectral data.

### 2.4 Spectral data preprocessing

After spectral acquisition, all spectra were processed in Python using a standardized workflow to reduce instrumental noise, fluorescence background, and intensity variation. The procedure included three steps: baseline correction, smoothing, and intensity normalization.

Baseline correction was performed with asymmetric least squares (AsLS) smoothing to remove the strong fluorescence background from the plant samples. This method, based on iteratively reweighted least squares, is well suited to estimating and subtracting complex non-linear baselines. The parameters were set as follows: regularization parameter λ = 1 × 10^6^, asymmetry factor p = 0.001, and number of iterations = 10. A Savitzky–Golay (S-G) filter was then applied to suppress high-frequency random noise, using a window length of 17 and a polynomial order of 2 (Savitzky and Golay, 1964).

Min–Max normalization was finally used to scale spectral intensities to the range of [0, 1], which reduced absolute intensity differences arising from variation in measurement position and local substrate enhancement. The normalization was calculated as follows:


$$I_{norm}=\frac{I-I_{\text{min}}}{I_{\text{max}}-I_{\text{min}}}$$

where I_min_ and I_max_ denote the minimum and maximum intensity values of an individual spectrum, respectively. This step made the relative intensities of spectral features comparable across samples.

### 2.5 Construction of machine learning discriminant models

To assess the classification performance of the SERS spectra and to compare different algorithms for high-dimensional spectral data, this study employed three supervised methods commonly used in Raman and SERS analysis: principal component analysis–linear discriminant analysis (PCA-LDA), partial least squares–discriminant analysis (PLS-DA), and support vector machine (SVM). These methods were used to build classification models that distinguished healthy samples from ToBRFV-infected samples.

To limit overfitting caused by the strong similarity among spectra collected from the same plant, the dataset was split using a Group Shuffle Split scheme. Partitioning was performed at the plant level rather than the spectrum level, so spectra from a given plant could not appear in both the training and test sets. With a random seed of 2020, 80% of the plants were assigned to the training set for model fitting and parameter tuning, whereas the remaining 20% were held out as an independent test set to assess generalization.

To eliminate any risk of data leakage, the train/test split was performed strictly at the plant level rather than at the spectrum level: the three SERS spectra recorded from any given plant were assigned together either to the training set or to the test set, ensuring that no plant contributed spectra to both. The same plant-level grouping was enforced inside the five-fold stratified group cross-validation used for hyperparameter selection.

Hyperparameters for PCA-LDA, PLS-DA, and SVM were optimized within the training set by five-fold stratified group cross-validation. The number of principal components was tuned for PCA-LDA, the number of latent variables for PLS-DA, and the penalty parameter (C) together with the kernel parameter (γ) for SVM. This design improved the reliability of model evaluation while avoiding leakage between the training and validation data (Roberts et al., 2017; Rosenblatt et al., 2024).

## 3 Results and discussion

### 3.1 Preliminary analysis of tomato leaf spectra

**Figure 2:**
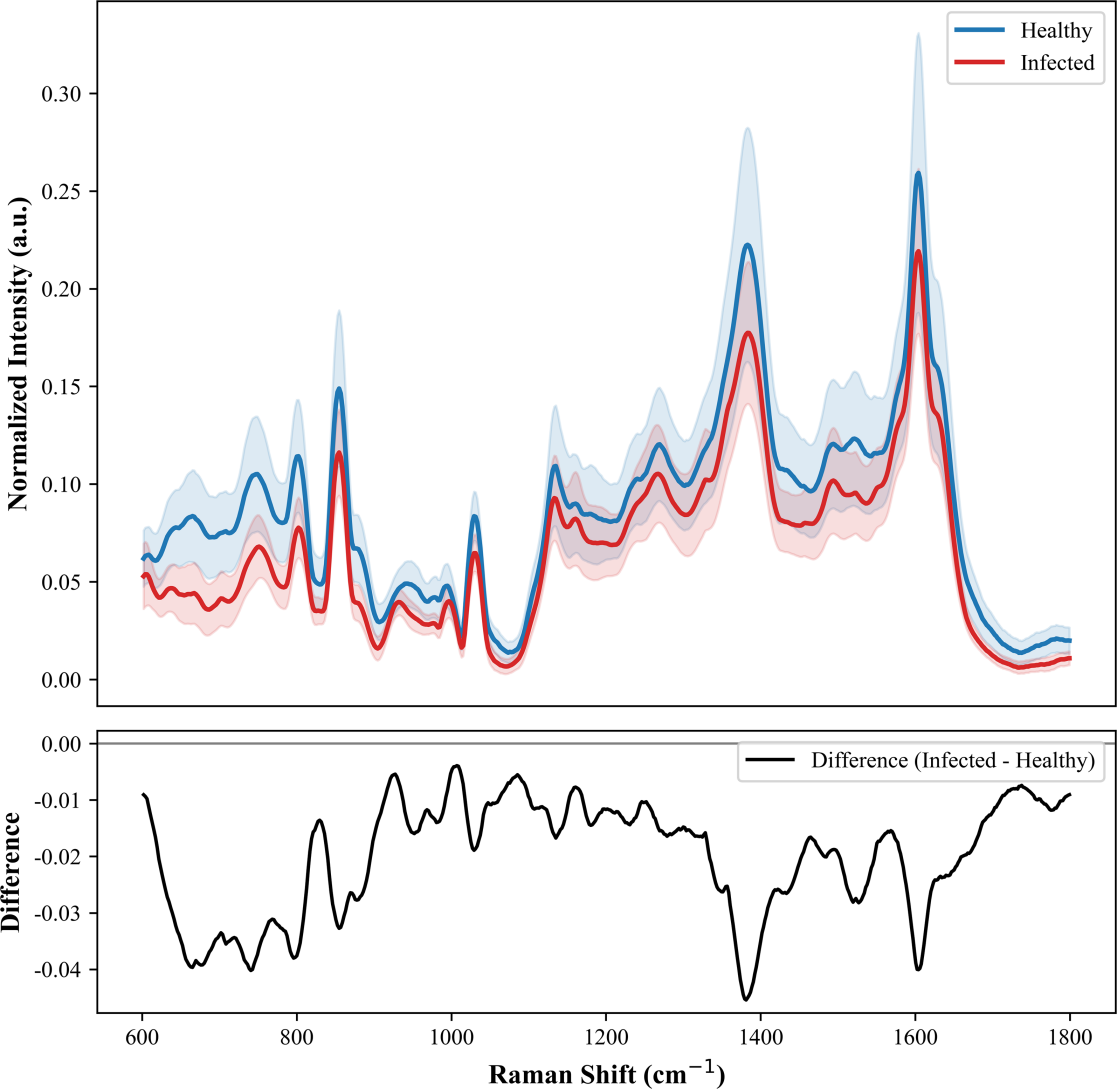
SERS spectra of leaf extracts from healthy and ToBRFV-infected tomato plants. The upper panel shows the mean normalized spectra over 600–1800 cm⁻¹; solid lines indicate mean intensity, and shaded areas indicate standard deviation. Blue represents healthy samples, and red represents infected samples. The lower panel shows the mean difference spectrum, calculated as the infected spectrum minus the healthy spectrum.

**Figure 2** presents the average preprocessed SERS spectra of healthy and ToBRFV-infected samples, along with the corresponding difference spectrum. To make the biochemical changes associated with viral infection easier to observe, the difference spectrum at the bottom marks the increases and decreases in the intensities of characteristic bands. Because SERS signals mainly originate from small molecules adsorbed on the silver substrate, ToBRFV infection may alter the composition and relative abundance of these metabolites. Furthermore, variations in the concentrations of these constituents may contribute to changes in the relative intensities of spectral peaks. This interpretation agrees with earlier reports showing that Raman spectroscopy can detect structural and concentration changes in plant biochemical components under pathogen stress, including carotenoids, pectin, and cellulose (Saletnik et al., 2024). The following discussion of the observed spectral changes is based primarily on the Raman band assignments summarized in Payne and Kurouski (2021) (Payne and Kurouski, 2021).

In the 600–800 cm⁻¹ region, the difference spectrum was predominantly negative and showed a broad low point near 747 cm⁻¹. Previous Raman assignments of plant macromolecules, especially pectin, attribute this band mainly to C–O–H vibrations of pectic carboxyl groups. The decreased signal intensity in this region might reflect a structural loosening of the tomato leaf cell wall. Accordingly, this spectral variation may be associated with a reduction in primary cell wall polysaccharides, particularly pectin, following infection with ToBRFV (Synytsya, 2003). Reviews of Raman studies on plant–pathogen interactions have likewise noted that pathogen invasion and systemic spread are often accompanied by cell-wall remodeling and enzymatic degradation of host structural barriers. This process provides a plausible explanation for the spectral differences observed between healthy and infected samples (Payne and Kurouski, 2021).

Several additional negative bands were observed at about 1387, 1515, and 1606 cm⁻¹, all showing clear attenuation. The largest decrease appeared near 1387 cm⁻¹. Published Raman assignments generally attribute this band to CH_3_ vibrations (Movasaghi et al., 2007). This pronounced decline may indicate that ToBRFV infection altered or reduced methyl-containing biochemical components in tomato leaf tissue. One possible reason is disruption of host lipid metabolism and membrane structure during viral replication. Positive-sense RNA viruses commonly reorganize host membrane systems to form membrane-associated replication compartments. Such changes may consume or redistribute lipid-related cellular constituents, which could explain the lower Raman intensity at this band (Zhang et al., 2019).

The infected samples also showed a marked reduction in carotenoid-related bands, especially in the 1510–1525 cm⁻¹, 1150–1170 cm⁻¹, and 1000–1020 cm⁻¹ regions. According to published Raman assignments for natural carotenoids, the bands near 1515 cm⁻¹ and 1155 cm⁻¹ are mainly assigned to C=C and C–C stretching of the carotenoid backbone, whereas the band around 1000–1020 cm⁻¹ is generally linked to methyl-related vibrations (Schulz et al., 2005, 2006). The major band near 1510 cm⁻¹ has been attributed chiefly to lycopene, while the band near 1520 cm⁻¹ has been associated with β-carotene (Baranska et al., 2006). In this study, these characteristic bands were clearly weaker in infected samples, indicating that ToBRFV infection altered carotenoid composition or relative abundance. The decrease in signal intensity may reflect not only pigment loss, but also stronger oxidative stress in infected tissues (Huseynova et al., 2018). Because viral replication can promote the accumulation of reactive oxygen species, carotenoids, which serve as essential antioxidants, may be consumed to protect membrane integrity and photosynthetic function. Consequently, this biochemical depletion likely contributed to the attenuated Raman intensities observed in this analysis (Hyodo et al., 2017; Sofy et al., 2021; Altangerel et al., 2017).

Another clear difference was observed near 1600 cm⁻¹, a region commonly assigned to aromatic ring vibrations of lignin, while the 1320–1330 cm⁻¹ region is generally related to CH_2_ bending (Agarwal, 2006). In the difference spectrum, the negative band at 1606 cm⁻¹ suggests that lignin- or phenylpropanoid-related components in tomato leaves were altered after ToBRFV infection. Earlier Raman studies of pathogen-stressed plant tissues have likewise shown that lignin-related bands near 1600 cm⁻¹ can weaken markedly or even disappear under infection, pointing to changes in lignin-associated cell-wall components (Farber and Kurouski, 2018). Since lignin forms an important structural barrier in the plant cell wall and contributes to resistance against pathogen invasion, the pronounced decrease at 1606 cm⁻¹ may indicate disturbance of cell-wall defense components or of phenylpropanoid metabolism during systemic ToBRFV infection (Miedes et al., 2014; Lee et al., 2019).

It is important to note that several of the bands that contribute most to discrimination — particularly the carotenoid (1155 and 1515 cm⁻¹), pectin (~747 cm⁻¹), and lignin (~1606 cm⁻¹) features — are general indicators of biotic and abiotic stress and have been reported in plants challenged with unrelated pathogens or with abiotic stressors such as salinity and nitrogen deficiency. The present binary classifier therefore detects a stress signature consistent with ToBRFV infection rather than a virus-specific molecular fingerprint. Discrimination is nonetheless achieved on the multivariate combinatorial pattern across these bands rather than on any single peak, which is the principle that allows SERS-ML to distinguish closely related metabolic states in cancer, microbiology, and plant pathology. Distinguishing ToBRFV from other tomato viruses, mechanical injury, nutrient deficiency, and abiotic stresses will require multi-class training data containing each of these arms; this work is in progress and forms the subject of a subsequent study.

The SERS spectral changes observed in this study likely reflect the multifaceted physiological and metabolic responses of the host plant induced by ToBRFV infection, rather than independent changes in a single metabolite. Plant viral infection may promote reactive oxygen species (ROS) accumulation and oxidative stress, thereby affecting chloroplast function, photosynthetic pigment metabolism, and antioxidant defense (Hyodo et al., 2017; Huseynova et al., 2018; Sofy et al., 2021). Because carotenoids participate in photoprotection and ROS scavenging, the attenuation of carotenoid-related SERS signals may be associated with virus-induced oxidative stress and changes in carotenoid composition or relative abundance (Altangerel et al., 2017).

As a positive-sense RNA virus, ToBRFV replication is likely accompanied by the remodeling of the host membrane system and the redistribution of membrane lipid components. Positive-sense RNA viruses commonly exploit host membrane structures to form membrane-associated replication compartments and manipulate host lipid metabolism to support viral RNA replication (Zhang et al., 2019). Therefore, the methyl- or lipid-related spectral changes near 1387 cm⁻¹ may be associated with virus replication-related membrane remodeling (Movasaghi et al., 2007; Zhang et al., 2019).

Furthermore, viral infection may induce cell wall remodeling and defense-related metabolic changes (Payne and Kurouski, 2021). The attenuation of pectin-related signals likely reflects alterations in the polysaccharide structure, modification status, or relative abundance of the primary cell wall during the infection process (Synytsya, 2003; Payne and Kurouski, 2021). Similarly, changes in lignin- and phenylpropanoid-related signals may signify modifications to the cell wall defense barrier and pathways associated with phenylpropanoid metabolism. Consequently, the variations in carotenoid-, pectin-, and lignin-related bands likely represent the collective contributions of ROS signaling, membrane remodeling, photosynthetic pigment perturbations, and cell wall defense responses following ToBRFV infection (Miedes et al., 2014; Farber and Kurouski, 2018). However, because the present study was based on SERS spectral assignments rather than targeted metabolomic or transcriptomic measurements, these pathway-level interpretations should be regarded as plausible mechanistic hypotheses rather than direct experimental confirmation of specific regulatory genes, enzymes, or metabolic fluxes.

Recent research on cell-type-specific plant stress responses provides an important conceptual framework for interpreting the spectral variability observed in this study. Ali et al. (2026) noted that traditional tissue-level analyses may mask response heterogeneity among different cell types, whereas single-cell and single-nucleus RNA sequencing and spatial transcriptomics can resolve plant responses to pathogen invasion and abiotic stress at both cellular and spatial resolutions. Their study also emphasized that plant stress and immune responses are not uniformly distributed across the entire tissue but are governed by spatial regulatory architecture, cell-type-specific stress perception, and intercellular signaling modules involving Ca²^+^, ROS, and phytohormones (Ali et al., 2026). Therefore, the SERS spectra obtained from whole-leaf extracts in the present study may represent integrated biochemical signals across diverse cell types and tissue regions, rather than signals from a discrete cell type or a single metabolic pathway. This perspective may help explain part of the spectral variability observed among infected samples. Future research could combine SERS with spatial transcriptomics, single-cell and single-nucleus transcriptomics, and spatial imaging techniques to further clarify the cell-type and spatial origins of SERS features.

In future studies, SERS could be further combined with metabolomics or transcriptomics to strengthen the biological interpretation of the spectral differences observed in this work. Metabolomics may help examine whether the biochemical components inferred from SERS band assignments show corresponding metabolite-level changes, while transcriptomics may provide additional information on stress- and defense-related gene-expression responses after ToBRFV infection. Such integrative analyses would provide complementary molecular evidence for interpreting SERS spectral variations and may help further confirm the biochemical basis of this SERS-based screening method.

### 3.2 Multivariate data analysis and spectral classification

SERS spectra are high dimensional and often exhibit strong correlations among spectral bands. After preprocessing, the data were analyzed with three machine learning models commonly used for binary classification in Raman and SERS studies. Their performance on the independent test set is summarized in **Table 1**.

**Table 1:** Performance of the PCA-LDA, PLS-DA, and SVM models on the independent test set. Evaluation metrics included accuracy, sensitivity, specificity, and area under the curve (AUC).

Model	Accuracy	Sensitivity	Specificity	AUC
PCA-LDA	88.33%	93.94%	81.48%	0.981
PLS-DA	90.00%	93.94%	85.19%	0.978
SVM	91.67%	100.00%	81.48%	0.993

#### 3.2.1 PCA-LDA

Raw SERS spectra are often high dimensional and may contain strong collinearity among wavenumber variables, baseline drift, and noise. Direct use of all wavenumber variables for classification can increase overfitting and weaken model generalization. PCA-LDA addresses these challenges by combining unsupervised dimensionality reduction with supervised classification. High-dimensional spectral data are first projected into a lower-dimensional feature space before the final classification step. This approach extracts principal components that capture the major sources of spectral variance, thereby reducing redundancy while preserving chemically relevant information (Jarvis and Goodacre, 2004; Lasalvia et al., 2022).

Principal component analysis (PCA) was first employed to reduce dimensionality and alleviate collinearity by projecting the data onto directions of maximal variance. This step also aided exploratory analysis and suppressed part of the noise. Linear discriminant analysis (LDA) was then performed within the reduced principal component space to identify projection directions maximizing linear separation between groups. Owing to its low computational burden and straightforward interpretation, PCA-LDA is well suited to small spectroscopic datasets (Quarin et al., 2025). Previous research demonstrates its value in SERS-based classification, including plant pathogen discrimination and the early detection of asymptomatic infections (Pérez et al., 2018; Vallejo-Pérez et al., 2021).

**Figure 3:**
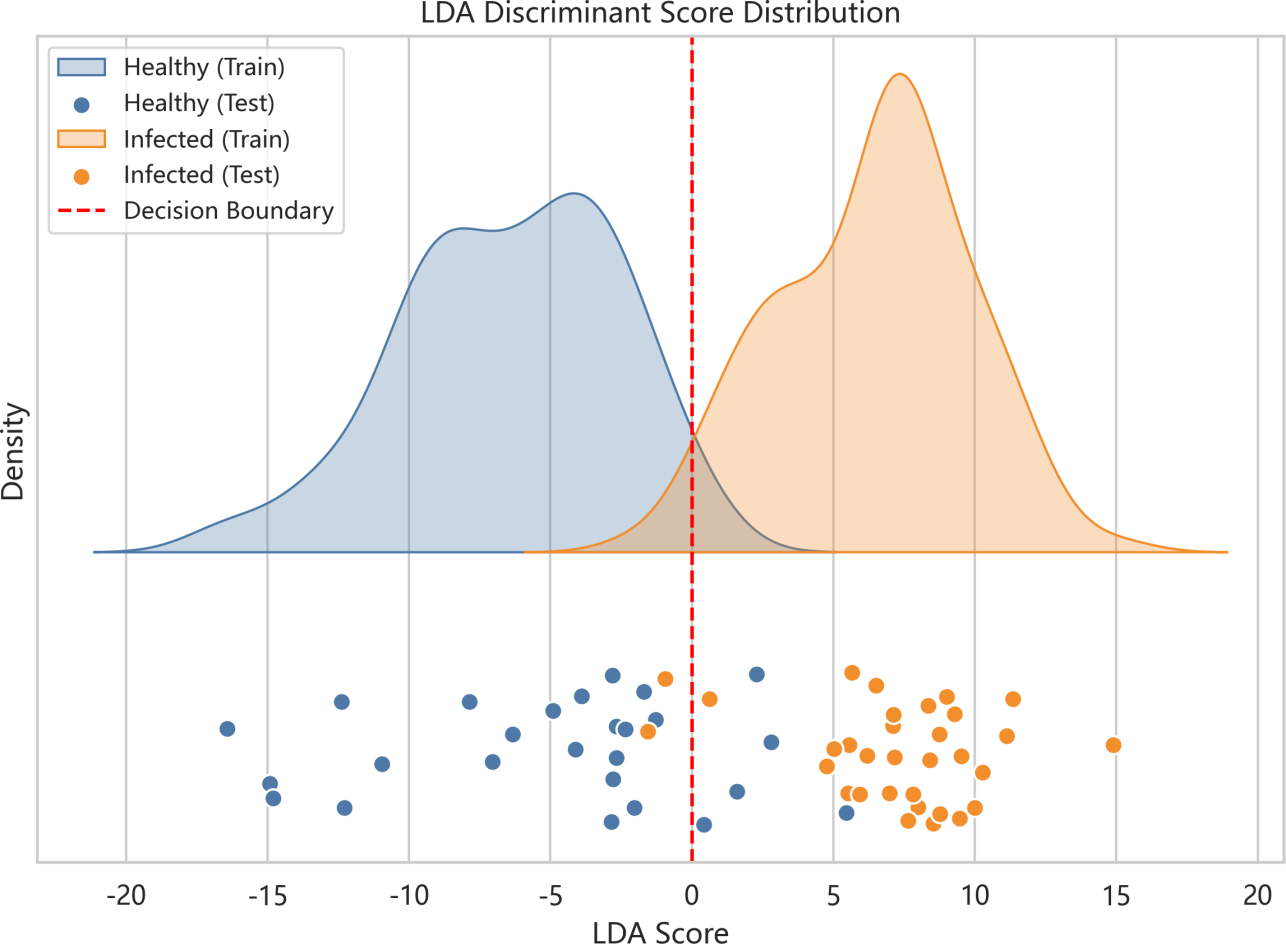
Distribution of LDA discriminant scores. The upper curves show the density distributions of healthy and infected samples in the training set, whereas the points below represent individual samples in the independent test set. The red dashed line at x = 0 marks the classification boundary.

PCA-LDA served as the first linear classifier for rapid discrimination in this study. The number of principal components was screened from 2 to 15 to retain informative variation while limiting noise. Five-fold grouped cross-validation on the training set determined that 12 principal components were optimal, accounting for 96.59% of the total variance. The LDA model was fitted with the default SVD solver. On the independent test set, PCA-LDA achieved an overall accuracy of 88.33% (**Table 1**). Sensitivity for infected samples reached 93.94%, and only two infected spectra were assigned to the healthy class. Specificity for healthy samples was lower, at 81.48%, with five healthy spectra misclassified as infected. These results indicate that some overlap remained between the spectral profiles of the two groups. **Figure 3** shows the same pattern: despite partial overlap, most test samples fell on opposite sides of the decision boundary (score = 0).

#### 3.2.2 PLS-DA

To make fuller use of class information and to address spectral collinearity, PLS-DA was examined as a second classification model.

PLS-DA constructs a limited number of latent variables that maximize the covariance between the spectral matrix X and the class response Y, while reducing data dimensionality at the same time. This makes the model more stable and improves class separation in small, high-dimensional datasets. PLS-DA also provides regression coefficients, loadings, and variable importance in projection (VIP) scores, which help identify the wavenumber regions most relevant to classification and link model output to characteristic SERS bands (Ballabio and Consonni, 2013). Previous SERS studies have also shown that PLS-DA can distinguish healthy plant samples from diseased samples and support early detection of pathogen infection (Mandrile et al., 2019; Sanchez et al., 2020b). Given the large number of spectral variables and the limited sample size in this study, PLS-DA was selected as the second linear model after PCA-LDA.

For PLS-DA, the number of latent variables (LVs) was optimized by five-fold grouped cross-validation within the training set. Model performance under different LV settings was evaluated using the root mean square error of cross-validation (RMSECV), while average classification accuracy was additionally monitored as a supplementary indicator. The optimal number of latent variables was determined according to the minimum RMSECV, and seven latent variables were selected. This setting preserved spectral differences between healthy and infected samples while limiting the inclusion of noise.

**Figure 4:**
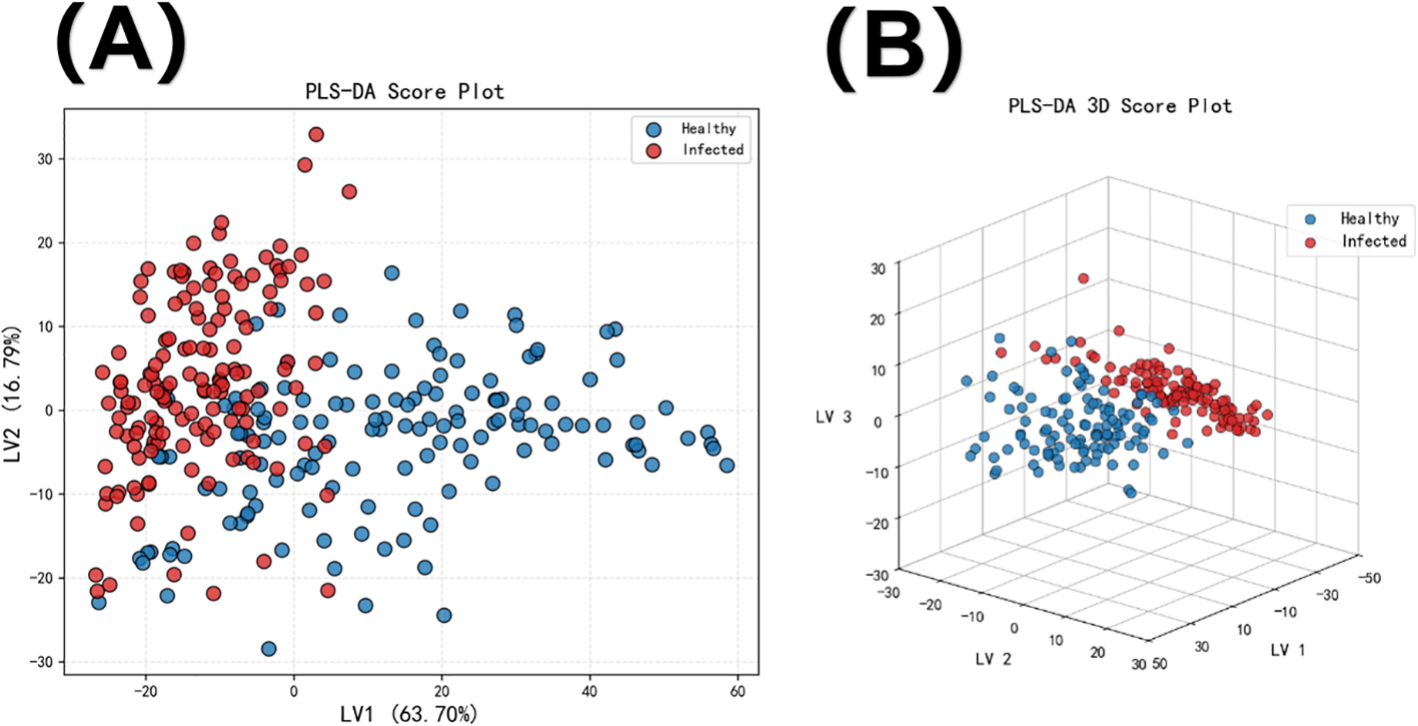
PLS-DA score plots for the training set, showing the separation between healthy and ToBRFV-infected samples. Healthy samples are shown as blue spheres, and infected samples as red spheres. **(A)** Two-dimensional score plot based on the first two latent variables (LV1 and LV2). **(B)** Three-dimensional score plot based on the first three latent variables (LV1, LV2, and LV3).

As shown in **Figure 4A**, the score plot of the first two latent variables (LV1 and LV2) explained 80.49% of the total spectral variance. Blue points denote healthy samples, whereas red points denote ToBRFV-infected samples. The distinct separation between the two groups suggests that viral infection induced measurable spectral modifications. PLS-DA improved the overall accuracy on the independent test set from 88.33% to 90.00% compared to PCA-LDA. This improvement primarily resulted from better identification of healthy samples. Specificity increased from 81.48% to 85.19%. Consequently, fewer healthy samples were misclassified as infected. Sensitivity for infected samples remained stable at 93.94%. The first three latent variables displayed a clearer group structure in three-dimensional space, as shown in **Figure 4B**. These results might imply that PLS-DA captured spectral features more closely linked to biological changes. This model potentially reduced the influence of background variation on the classification performance.

#### 3.2.3 SVM

Biological SERS spectra often contain complex nonlinear features that linear models, such as PCA-LDA and PLS-DA, may not fully capture. We therefore evaluated support vector machine (SVM) as an additional classifier. SVM is well suited to small, high-dimensional datasets and can handle non-linear classification effectively. Unlike PCA-LDA and PLS-DA, which seek optimal linear projection directions, SVM uses a kernel function to map spectral data into a higher-dimensional feature space, where non-linear class boundaries can be resolved more effectively. Earlier SERS studies have likewise shown that SVM performs well when sample groups overlap in low-dimensional space (Noble, 2006; Wang et al., 2025a). In this study, SVM was used to further assess the non-linear discriminative potential of the spectral data.

Consistent with the previous models, the dataset was divided into a training set and a held-out test set at a ratio of 4:1. Within the training set, the penalty parameter C and kernel parameter γ were optimized by grid search combined with five-fold grouped cross-validation. The final SVM model was then built with C = 200 and γ = 0.0001 using a radial basis function (RBF) kernel.

**Figure 5:**
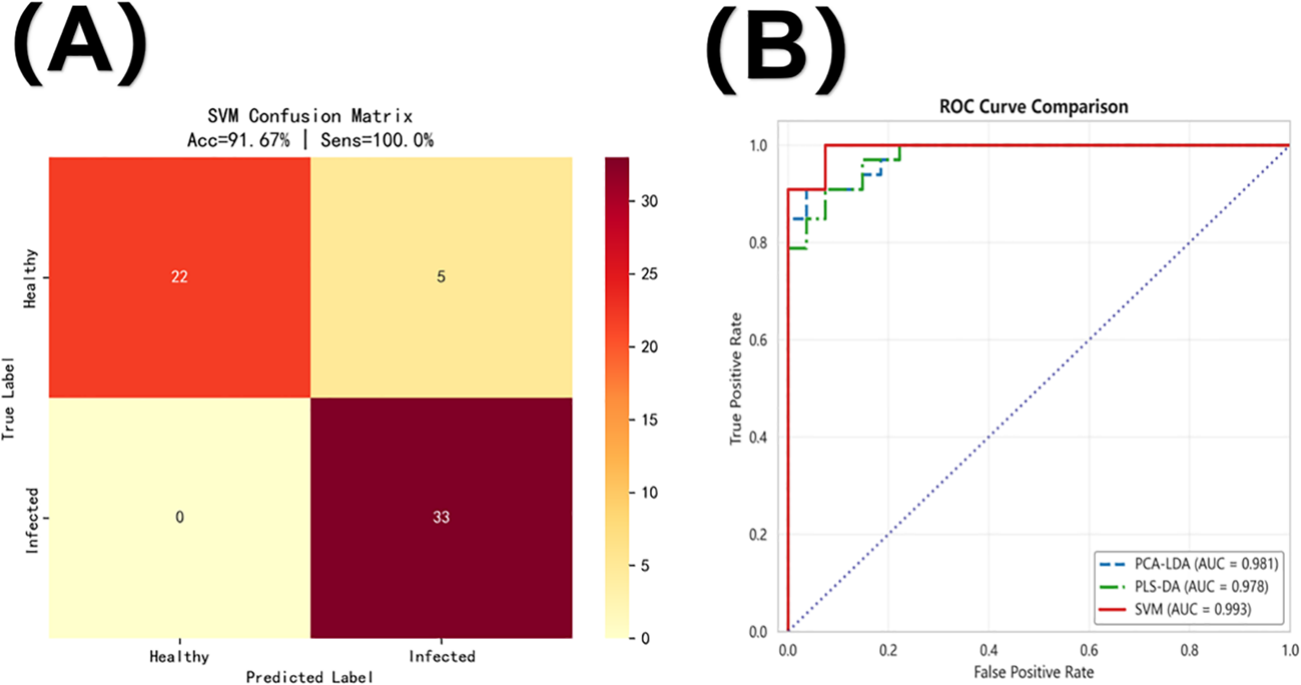
Classification performance of machine learning models on the independent test set. **(A)** Classification results of the SVM model. **(B)** ROC curves of the PCA-LDA, PLS-DA, and SVM models.

**Figure 5A** shows the SVM classification results on the independent test set. The model achieved an overall accuracy of 91.67%. Sensitivity for infected samples reached 100.00%, indicating strong screening ability for ToBRFV infection. **Figure 5B** shows the ROC curves of the three models on the test set. The SVM model yielded an AUC of 0.993, suggesting strong discrimination performance within the current test set, although a small number of false positive predictions remained.

#### 3.2.4 Sensitivity/specificity trade-off and integration into a diagnostic workflow

The SVM model achieved 100% sensitivity and 81.48% specificity on the independent test set. This asymmetric operating point is consistent with the intended use of the method as a preliminary screening tool. ToBRFV is highly transmissible — mechanically and via seeds — and is capable of overcoming Tm-2² resistance; therefore, the epidemiological cost of a missed infection in a commercial greenhouse may exceed the cost of an unnecessary confirmatory test. Under such cost asymmetry, screening workflows often prioritize high sensitivity to reduce the risk of false-negative results.

To illustrate the practical implications of this trade-off, we considered a hypothetical commercial greenhouse scenario with N = 10,000 tomato plants and a low background ToBRFV prevalence of 1% (100 truly infected and 9,900 truly healthy). Assuming that the test-set sensitivity of 100% and specificity of 81.48% were maintained in this scenario, the resulting confusion matrix would yield 100 true positives, 0 false negatives, 8,067 true negatives, and 1,833 false positives. From these:

- Negative Predictive Value (NPV) = 100% — no infected plants would be missed under this illustrative assumption.
- Positive Predictive Value (PPV) = 100/(100 + 1,833) ≈ 5.17%.
- Total plants requiring RT-qPCR confirmation = 1,933.
- Reduction in molecular-testing burden ≈ 80.67% (from 10,000 to 1,933 RT-qPCR runs).

In other words, under this hypothetical scenario, the proposed two-tier workflow would eliminate approximately four out of every five RT-qPCR runs that would otherwise be needed for exhaustive surveillance, while maintaining all infected plants in the confirmatory testing stream under the assumed model performance. The same calculation at higher prevalence values yields PPVs of ≈ 21.4% at 5% prevalence and ≈ 37.5% at 10% prevalence, with NPV remaining at 100% under the same sensitivity assumption.

We therefore propose a two-tier diagnostic workflow: rapid SERS-ML screening of candidate plants, followed by RT-qPCR confirmation of every plant flagged positive by the screen, with management decisions taken exclusively on the basis of the RT-qPCR result. This positions SERS-ML as a triage step that may reduce molecular-testing burden without compromising the diagnostic authority of RT-qPCR. The decision threshold of the classifier could additionally be adjusted in future externally validated implementations: a more conservative threshold may be suitable for production-stage screening, where the cost of false positives is greater, whereas a maximum-sensitivity threshold may be preferable in quarantine settings, where missing an infected plant is unacceptable.

### 3.3 Limitations and future perspectives

Although SERS combined with machine learning shows potential for rapid, label-free preliminary screening of ToBRFV, the present study remains an initial proof of concept based on RT-qPCR-confirmed, mechanically inoculated plants. Several limitations must be acknowledged and are discussed below.

#### 3.3.1 Substrate reproducibility and SERS measurement stability

The reproducibility and stability of SERS substrates remain important technical factors affecting the practical application of this method. In this study, Ag nanorod arrays fabricated on silicon wafers by glancing angle deposition (GLAD) were used as the SERS-active substrates. Previous studies have shown that this method can produce large-area Ag nanorod array substrates with relatively uniform morphology, and the substrate fabrication in the present study followed parameters reported in published work. However, this study did not systematically evaluate substrate reproducibility across different fabrication batches, long-term storage stability, or differences in enhancement performance across different regions of the substrate. Although three spectra were collected from different positions on the substrate for each sample, and Min–Max normalization was applied to reduce the effects of local enhancement differences and measurement-position variation, these measures mainly improved spectral comparability within the current dataset and cannot replace systematic validation of intra-batch and inter-batch substrate reproducibility.

#### 3.3.2 Environmental noise and sample-background interference

Environmental noise and complex sample backgrounds may also affect the stability of SERS spectra and the generalizability of the model. Under real agricultural conditions, factors such as temperature, humidity, light exposure, plant water status, leaf surface contaminants, pesticide residues, dust, and other abiotic stresses may alter the metabolic composition and relative abundance of biochemical constituents in tomato leaves, or introduce additional background interference. In this study, ethanol extraction and 100-fold dilution were used to reduce matrix effects from plant extracts, while baseline correction, smoothing, and normalization were applied to reduce spectral noise and intensity variation. However, these procedures are mainly applicable to the controlled experimental samples used in the present study and do not fully represent the interference resistance of the method under complex field conditions.

#### 3.3.3 Diagnostic specificity and biological complexity

Biological and ecological complexity in real agricultural production may further influence the applicability and diagnostic specificity of the model. The present study was mainly limited to binary screening between healthy tomato leaves and leaves infected with ToBRFV, and the samples were obtained under relatively controlled experimental conditions. Therefore, the spectral differences identified here should be interpreted as ToBRFV-associated spectral features under the tested conditions, rather than exclusive ToBRFV-specific molecular biomarkers validated across diverse field environments.

The SERS spectral changes observed in this study are more likely to reflect physiological and biochemical responses of the host plant to viral infection — including attenuation of carotenoid signals associated with oxidative stress and variations in pectin- and lignin-related signals linked to cell wall remodeling — rather than direct spectral signatures of the ToBRFV particles themselves. Similar biochemical changes may also occur under other biotic and abiotic stresses. The present approach relies on machine learning classification of high-dimensional combinatorial spectral patterns across the full spectrum, which may help capture a more comprehensive infection-associated metabolic fingerprint. The diagnostic specificity of this approach therefore requires further validation with more complex sample systems.

#### 3.3.4 Field applicability and early-stage detection

It is important to note that the infected samples in this study were obtained at a fixed sampling time point under controlled experimental conditions. Specifically, they were collected at 14 days post-inoculation, and their infection status was confirmed by RT-qPCR. Therefore, the current findings cannot directly demonstrate the reliability of this method for detecting asymptomatic or very early-stage infections under field conditions.

#### 3.3.5 Model generalizability and validation strategy

Furthermore, plant-level data splitting and grouped cross-validation were used during model construction. This approach reduces the risk of data leakage and overfitting arising from multiple spectra collected from the same plant. Nevertheless, it primarily assesses internal classification stability within the current dataset and cannot substitute for independent external validation across diverse cultivars, developmental stages, and environmental conditions.

#### 3.3.6 Co-infection and mixed-pathogen scenarios

Because the present study used a binary classification design to distinguish healthy tomato leaves from leaves infected with ToBRFV, it cannot directly determine whether SERS can distinguish co-infections or mixed-pathogen scenarios commonly encountered in agricultural production. Under real agricultural conditions, multiple pathogens may coexist and induce overlapping or combined host biochemical responses, thereby increasing the complexity of SERS spectral interpretation and model classification.

#### 3.3.7 Validation studies in progress and planned

All tomato plants used in this study were of a single cultivar, grown under controlled greenhouse conditions, with infection established by mechanical inoculation rather than natural transmission. Field-grown plants are routinely subject to cultivar variation, fluctuating light and temperature, irrigation regimes, nutritional imbalances, and the possibility of mixed infections, all of which can modulate carotenoid, pectin, and phenylpropanoid metabolism — i.e. the very biochemical compartments that contribute to the discriminating bands identified here. The 91.67% accuracy reported here should therefore be interpreted as an upper bound under controlled conditions rather than as a generalized performance estimate.

Validation on naturally infected, field-collected samples with parallel RT-qPCR confirmation is required before practical deployment. To this end, an ongoing multi-site, multi-cultivar campaign is currently being established in collaboration with local agricultural extension services. The campaign covers paired healthy/infected samples from at least two commercial greenhouses across the spring and autumn cropping cycles, three commonly grown cultivars, parallel RT-qPCR confirmation for every sample, and a leave-one-source-out cross-validation strategy to assess transferability across diverse agricultural environments. Because ToBRFV is a regulated quarantine pathogen in our region, this campaign is conducted under biosafety containment and is necessarily a multi-season undertaking.

A second axis of future work concerns specificity. The bands that drive discrimination here are general indicators of biotic and abiotic stress and are not unique to ToBRFV. A four-arm specificity-validation extension is therefore planned, comprising (i) other tomato tobamoviruses (TMV, ToMV); (ii) Tomato yellow leaf curl virus (a begomovirus, distinct genome class); (iii) abiotic stress controls (drought, salinity, and nitrogen deficiency); and (iv) mechanically injured but uninfected leaves. A multi-class classifier trained on these arms will be the natural next step toward a virus-specific assay.

## 4 Conclusions

This study developed a label-free method for rapid preliminary screening of Tomato brown rugose fruit virus (ToBRFV) by combining surface-enhanced Raman scattering (SERS) with machine learning. The spectra suggested that viral infection may alter the biochemical profile of tomato leaves, with the most notable changes appearing in bands assigned to carotenoids and pectin. These features may provide a biochemical basis for future screening studies at earlier infection time points, although validation using asymptomatic samples, field-collected materials, and independent external datasets remains necessary. During model development, the data were split at the plant level rather than the spectrum level. This reduced the risk of data leakage and provided a more conservative estimate of internal classification performance. Among the tested models, SVM showed the best performance, with an accuracy of 91.67% and very high sensitivity for infected samples, which is especially important for ToBRFV screening because of the rapid spread and high transmissibility of the virus. Compared with RT-qPCR, the SERS method requires simpler sample preparation and may be more suitable for rapid preliminary screening. At present, the method is limited to binary screening under relatively controlled conditions, and further validation is needed before direct deployment because real agricultural settings may involve abiotic stresses and co-infections that introduce complex background interference into the SERS spectra. We emphasize that, in its present form, this approach is intended as a rapid, low-cost, field-deployable preliminary screening/triage tool, not as a replacement for RT-qPCR or other confirmatory molecular assays. RT-qPCR remains the indispensable reference standard for plant quarantine and official disease diagnosis; the SERS-ML workflow proposed here is positioned upstream of RT-qPCR, where it can substantially reduce the molecular-testing burden without compromising biosecurity. Demonstrated robustness across cultivars, growth stages, environmental stresses, and co-infecting pathogens, together with multi-class specificity validation against other tomato viruses and abiotic stresses, will be required before clinical or quarantine deployment, and forms the focus of our ongoing follow-up work.

## Data Availability

The raw data supporting the conclusions of this article will be made available by the authors, without undue reservation.
